# Activated fibroblasts in muscle sarcoidosis revealed by [^18^F]FAPI-74 PET

**DOI:** 10.1007/s00259-023-06263-8

**Published:** 2023-06-05

**Authors:** Tadashi Watabe, Takahito Fukusumi, Hidenori Inohara, Mitsuaki Tatsumi, Sadahiro Naka, Takashi Kamiya, Hiroki Kato, Yuriko Mori, Jens Cardinale, Frederik L. Giesel

**Affiliations:** 1 Department of Nuclear Medicine and Tracer Kinetics, Graduate School of Medicine, Osaka University, 2-2 Yamadaoka, Suita, Osaka, 565-0871 Japan; 2 Institute for Radiation Sciences, Osaka University, Osaka, Japan; 3 Department of Otorhinolaryngology-Head and Neck Surgery, Graduate School of Medicine, Osaka University, Osaka, Japan; 4 Department of Radiology, Osaka University Hospital, Osaka, Japan; 5 Department of Pharmacy, Osaka University Hospital, Osaka, Japan; 6 Department of Nuclear Medicine, Medical Faculty, University Hospital Duesseldorf, Heinrich-Heine-University, Dusseldorf, Germany

A 73-year-old female patient with papillary thyroid cancer underwent ^18^F-fluorodeoxyglucose ([^18^F]FDG) and ^18^F-fibroblast activation protein inhibitor-74 ([^18^F]FAPI-74) positron emission tomography (PET) for the evaluation of metastatic lesions prior to surgery. She had been diagnosed with sarcoidosis histologically using biopsies from the mediastinal lymph node and skin 7 years before, and CT scan prior to PET scan showed enlargement of the mediastinal lymph nodes. [^18^F]FDG PET showed increased uptake in the mediastinal lymph nodes, suggestive of reactive uptake, or metastasis (Fig. A and B: maximum intensity projection (MIP) and axial fusion). Additionally, mild uptake was observed in the diaphragm and oblique muscles. However, the lower extremities were not included in the scanned area, as no abnormal accumulation was found in the proximal thigh within the normal imaging range from the top of the head to the mid-thigh. On [^18^F]FAPI-74 PET, multiple regions of abnormal uptake were observed in the lower extremity muscles (Fig. C and D: MIP and coronal PET and fusion, red arrows). These abnormal uptakes exhibited a linear or bar-shaped pattern along the muscular striations (SUVmax = 7.32). Mild linear accumulation was also observed in the diaphragm, and increased uptake was also detected in the paraspinal muscles (Fig. C and E: MIP and axial PET and fusion, red arrows). The patient has been followed up with the diagnosis of sarcoidosis in the lungs, skin, and eyes. Based on her history and muscle uptake patterns, she was diagnosed with muscular sarcoidosis (asymptomatic sarcoid myopathy).

According to previous studies, the ‘leopard-man sign’ or ‘tiger-man sign’ is observed on ^67^Ga scintigraphy and [^18^F]-FDG PET among patients with sarcoid myopathy [1, 2].

Asymptomatic myopathy occurs in 50–80% of patients with sarcoidosis, while less than 5% of patients have clinical symptoms [2]. Marie et al. observed multiple linear uptake patterns on [^18^F]-FDG in the calf and thigh muscles of both legs in a patient with biopsy-proven pulmonary and muscular sarcoidosis [3]. This was considered the typical pattern for sarcoid myopathy, and its imaging finding is similar to that observed on [^18^F]FAPI-74 PET of the present case despite the difference of being asymptomatic without any increase in white blood cell count or C-reactive protein. Cardiac sarcoidosis exhibits increased uptake on [^68^Ga]FAPI-46 PET without increased uptake on [^18^F]FDG PET. This corresponded to the lesions that exhibited late gadolinium enhancement on magnetic resonance imaging, suggestive of activated fibroblasts in the fibrotic remodelling processes [4]. However, there have been no previous reports on muscle sarcoidosis, presenting with increased uptake on FAPI-PET. To the best of our knowledge, this is the first reported case of activated fibroblasts in sarcoid myopathy. A comparison of MIP images between FAPI-PET and FDG-PET showed high uptake in the paraspinal muscle on FAPI-PET (topmost red arrows in Fig. C), whereas no abnormal accumulation was observed on FDG-PET. We assume that the muscle uptake on FAPI-PET might be due to fibrotic changes by activated fibroblasts in the chronic phase, independent of the active inflammation detectable on FDG-PET since the patient was asymptomatic and had been stable for years without further treatment. The potential limitation of the present case report is the lack of histological confirmation of sarcoid myopathy, as the biopsy was not justified for the asymptomatic patient. Chronic muscular sarcoidosis should be suspected when the typical pattern of multiple muscle uptake is observed on FAPI-PET.


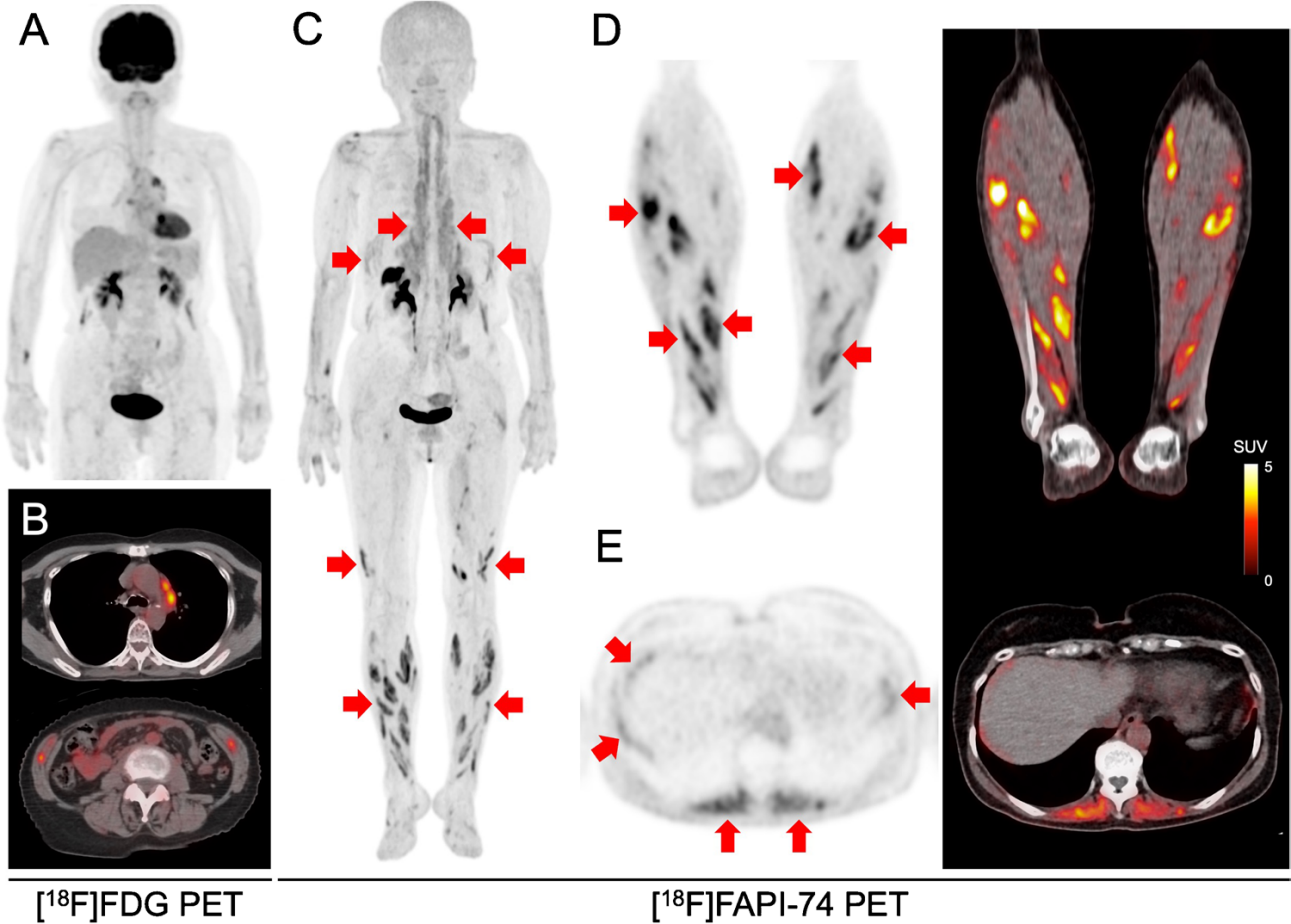


## Data Availability

Data sharing not applicable to this article as no datasets were generated or analysed during the current study.
